# Micro-Imaging by Interference Microscopy: A Case Study of Orientation-Dependent Guest Diffusion in MFI-Type Zeolite Host Crystals

**DOI:** 10.3390/ma5040721

**Published:** 2012-04-24

**Authors:** Laurent Gueudré, Tomas Binder, Christian Chmelik, Florian Hibbe, Douglas M. Ruthven, Jörg Kärger

**Affiliations:** 1 Department of Interface Physics, University of Leipzig, Leipzig 04109, Germany; E-Mails: laurent.gueudre@uni-leipzig.de (L.G.); tomas@uni-leipzig.de (T.B.); chmelik@physik.uni-leipzig.de (C.C.); hibbe@physik.uni-leipzig.de (F.H.); 2 Department of Chemical and Biological Engineering, University of Maine, Orono, ME 04473, USA; E-Mail: druthven@umche.maine.edu

**Keywords:** MFI (mordenite framework inverted), diffusion, anisotropy, surface resistance, interference microscopy

## Abstract

Because of the small particle size, orientation-dependent diffusion measurements in microporous materials remains a challenging task. We highlight here the potential of micro-imaging by interference microscopy in a case study with MFI-type crystals in which, although with different accuracies, transient concentration profiles in all three directions can be observed. The measurements, which were performed with “rounded-boat” shaped crystals, reproduce the evolution patterns of the guest profiles recorded in previous studies with the more common “coffin-shaped” MFI crystals. The uptake and release patterns through the four principal faces (which in the coffin-shaped crystals extend in the longitudinal direction) are essentially coincident and there is no perceptible mass transfer in the direction of the long axis. The surface resistances of the four crystal faces through which mass transfer occurs are relatively small and have only a minor effect on the mass transfer rate. As a result of the pore structure, diffusion in the crystallographic *c* direction (which corresponds to the direction of the long axis) is expected to be much slower than in the transverse directions. This could explain the very low rate of mass transfer observed in the direction of the long axis, but it is also possible that the small end faces of the crystal may have high surface resistance. It is not possible to distinguish unequivocally between these two possibilities. All guest molecules studied (methyl-butane, benzene and 4-methyl-2-pentyne) show the same orientation dependence of mass transfer. The long 4-methyl-2-pentyne molecules would be expected to propagate at very different rates through the straight and sinusoidal channels. The coinciding patterns for uptake through the mutually perpendicular crystal faces therefore provide clear evidence that both the coffin shaped crystals and the rounded-boat-shaped crystals considered in this study, must be intergrowths rather than pure single crystals.

## 1. Introduction

Mass transfer is critically important for most of the technological applications of nanoporous materials, including separation, catalysis, gas storage and sensing applications [1,2,3,4,5]. The exploration of mass transfer rates for confined molecules is among the hot topics of current fundamental research [6,7,8]. In many nanoporous materials the crystal structure is non-isotropic (non-cubic). Structural anisotropy immediately gives rise to anisotropy in the guest mobilities. Consequently, mass transfer in such materials must be characterized by a diffusion tensor, *i.e.*, by three principal values and the orientation of the tensor main axes, rather than by a single diffusivity. Since the positions of the atoms forming the host lattice of such materials are known from X-ray diffraction [9,10,11] and since there exist well established approaches for modeling the force field exerted on the guest molecules by both the host lattice and other guest molecules [12,13,14,15], the exploration of diffusion anisotropy has become a popular area for molecular dynamics simulations, especially for zeolites of structure types MFI [16,17,18,19,20,21,22,23] and CHA [24,25].

In addition to attempts to predict the propagation rates in different directions from molecular dynamics simulations, an alternative approach has been developed, based on structural considerations that suggest that, in some nanoporous crystalline materials, the pore geometry should give rise to well-defined interdependences between the rates of mass transfer in the different crystallographic directions. Prominent examples of host systems in which this phenomenon of “structure-correlated diffusion anisotropy” may be expected to occur include the zeolites of type MFI [26,27] and CHA [28]. Also here, corroboration from molecular dynamics simulations turned out to be most valuable for determining the conditions under which the resulting correlation rules are applicable. For example, in such simulations, the correlation rules were found to hold for small hydrocarbons in MFI [16,20], including even for multicomponent diffusion [21], while for water in chabazite [24,25] or long-chain paraffins in MFI [23], due to specific host-guest interactions, deviations from these simple rules are to be expected.

It is important to note, however, that these detailed predictions from molecular dynamics simulations of diffusion anisotropy in zeolites have generally not been confirmed by experimental studies. This is particularly true for MFI-type zeolites which generally have a twin structure, *i.e.*, the crystals are intergrowths rather than genuine single crystals. Kocirik and co-workers [29] confirmed the form of this sub-structure by demonstrating that iodine distributes rapidly along the interfaces between the different sub-sections. After treating with alkaline hydrogen peroxide solution at elevated temperatures and ultrasound, Schmidt *et al.* [30] were able to break the intergrowths into their individual segments, which showed a coherent crystal structure [31].

Interestingly, the deviations from ideal single-crystalline morphology obviously did not affect the first pulsed field gradient nuclear magnetic resonance (PFG NMR) studies of diffusion anisotropy [32,33]. Analysis of the measurements on the basis of the correlation rule of diffusion anisotropy in MFI-type zeolites [26,27] yielded self-consistent results. This may be understood by realizing that the diffusion path lengths covered in these studies were of the order of only a few micrometers. Therefore most of the observed diffusion paths remained within the same subunit, which is effectively a structurally homogeneous single crystal.

The most important conclusion from these early PFG NMR studies is that diffusion in the direction of the longitudinal extension of the crystals was found to be much slower than in the perpendicular directions. This finding is consistent with the general assumption that, in the typically coffin-shaped MFI-type crystals, the channel pores (the sinusoidal and straight channels) are directed perpendicular to the longitudinal crystal extension so that transport in the longitudinal direction has to occur by alternating periods of travel through straight and sinusoidal segments. The required changes of direction at the channel intersections are rate-limiting and lead to a reduction in the diffusivity by a factor (referred to as the anisotropy factor) of about 5 [26].

Similarly, in uptake measurements with crystals embedded in sputtered copper [34], uptake in the transverse direction, *i.e.*, through the large crystal faces, was found to give rise to substantially larger diffusivities than uptake in the longitudinal direction (as observed for crystals embedded vertically). In this case, the anisotropy factor was found to be about three.

Orientation-dependent diffusivities may also be determined from single-crystal permeation studies [35,36,37]. In these measurements, single MFI-type crystals are embedded within an otherwise impermeable membrane. Since the crystals are oriented with their longitudinal extension perpendicular to the plane of the membrane, the permeation rate yields the diffusivity in that direction. For several systems for which comparative data are available, the mean diffusivity values derived from the membrane measurements do not differ significantly from the mean values of the diffusivities determined from macroscopic rate measurements (for example by the ZLC technique) [38,39,40,41] which measure the average diffusivity in all directions. There is a good deal of scatter but these data do not provide any real indication of the expected anisotropy.

Single-crystal micro-imaging by interference microscopy has provided us with a new tool for observing diffusion anisotropy. It makes it possible to follow the evolution of transient concentration profiles by recording the time dependence of the integral over local intracrystalline concentrations in the observation direction which, for crystals of constant thickness, may also be interpreted as the mean value of the intracrystalline concentration in the observation direction. In this way, one obtains two-dimensional maps of these concentration integrals (or: mean concentrations) and their variation with time. In a typical experiment, molecular uptake or release, in response to a well-defined pressure step, is followed. However, it is also possible to follow the response to any other variation in the external pressure, including the so-called partial loading experiments [42,43,44] where the first pressure step is followed by a second one which is applied before the crystal under study has equilibrated with the surrounding gas phase [45].

The measurement of integrals, rather than of local concentrations, does not impose any restriction in the viability of this technique provided that, in the system under study, mass transfer is confined to the directions perpendicular to the observation direction. In this case, the integral in the observation direction degenerates to the simple product of the local concentration and the crystal thickness. The excellent measuring conditions provided in such situations have been extensively exploited by considering nanoporous materials with pore systems extended in either one [45,46,47,48,49,50] or two [51,52] dimensions.

In three-dimensional pore networks, mass transfer generally occurs also in the observation direction. Under these conditions, the determination of local concentrations from the concentration integrals becomes an “ill-posed” problem. In very detailed studies with MFI type zeolites [53,54,55] the underlying diffusivities and transport resistances were determined by looking for the best fit between the experimentally-determined concentration integrals and corresponding solutions of the diffusion equation.

Information on the local concentrations becomes more reliably accessible if, in addition to the concentration integrals recorded during a given transient sorption experiment, the same experiment is repeated with the crystal in a different crystallographic orientation. The benefit of such studies has been demonstrated with zeolite crystals of type SAPO STA-7 [56,57] where local molecular concentrations during molecular uptake could be reliably extracted from the concentration integrals in two different observation directions.

As a result of similar studies with coffin-shaped MFI-type zeolites [58], the concentration integrals during molecular uptake were found to be essentially the same for any of the four possible positions in which the crystals could be placed within the adsorption cell, providing clear evidence that the individual zeolite particles, though appearing as single crystals, were twinned intergrowths.

From detailed studies using fluorescence microscopy [59,60], the intergrowth structure of MFI-type zeolites is known to depend on the conditions during crystallization. This finding suggests that there is no *a-priori* limitation that prohibits the synthesis of structurally coherent MFI-type crystals. Being able to measure orientation-dependent diffusivities, interference microscopy has a unique position among the techniques applicable in the search for such materials. As a case study, the present communication reports the results of micro-imaging studies with a class of MFI type crystals which, by following a procedure reported in references [61,62,63], may be synthesized as particles which allow the novel possibility of observing the concentration integrals in all three directions.

The benefit of these novel options and the associated surplus in information appears in an impressive diversity of the thus accessible transient concentration profiles of guest molecules during uptake and release illustrating, in unprecedented clarity, the interplay of the transport resistances in the intracrystalline pore space and on the crystal boundary, and the impact of their concentration dependences.

## 2. Experimental Section

### 2.1. Synthesis

Following the recipe published in [61], the MFI-type crystals used in this study were synthesized with a TPA-silicalite-1-precursor sol prepared by hydrolyzing a silicon source (Aerosil 130) with a structuring agent (tetrapropylammonium bromide) and a complexing agent (benzene-1,2-diol) in water, yielding the relation 60 SiO_2_/12 TPABr/15 NaOH/24 benzene-1,2-diol/1800 H_2_O. The sol was subsequently heated in an autoclave up to 433 K and kept there for 7 days without stirring. The hydrolyzed solution was filtered through a filter membrane (nominal pore size 0.2 µm). The template was removed from the micropore by calcination in O_2_/air mixture at 50/50 at 823 K for 12 h. No additional treatment (including surface leaching) was performed prior to the calcination.

**Figure 1:**
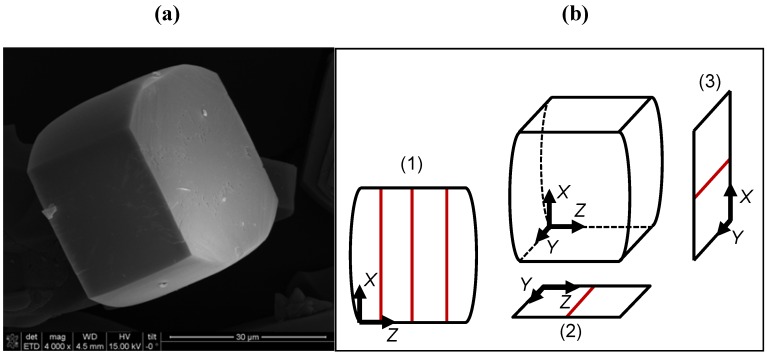
(**a**) SEM image of a typical crystal applied in this study and (**b**) the different orientations under which the concentration profiles during molecular uptake (Figure 2, Figure 3, Figure 4, Figure 5 and Figure 6) were recorded.

Figure 1a displays the scanning electron micrograph of a typical crystal obtained by this analysis. It is of the “rounded-boat” shape, well-known for this type of synthesis. The average crystal size is about 40 × 28 × 40 µm^3^. X-ray diffraction analysis confirmed the structure and showed that the sample was substantially free of amorphous silica. From the nitrogen isotherm at 77 K, the micropore volume was estimated to be close to a value of 0.18 cm^3^/g.

### 2.2. Micro-Imaging by Interference Microscopy

Detailed descriptions of the application of interference microscopy and of the way in which the primary data observable in the experiments are transferred to the relevant transport parameters may be found in the reviews [64,65,66,67] and in the text book [8]. In short, the application of interference microscopy to the observation of transient concentration profiles is based on the fact that the diffraction index, *i.e.*, the optical density and, hence, the optical path length for a light beam passing through a nanoporous crystal is, *inter alia*, a function of the local concentration. In first-order approximation the optical density can be assumed to vary linearly with the local concentration. Hence, any change in local concentration leads to changes in the optical path length and, hence, in the phase of the light beam passing through the crystal. These changes are recorded by observing the interference patterns with light passing through the surrounding atmosphere (which, for observation of interference, must be coherent with the light passing through the crystal). Changes in the interference patterns may therefore be related directly, by an appropriate computer program, to the changes in the concentration integral.

We refer to Figure 1 to visualize the attainable information. Considering that, within the optical (and sorption!) cell, the crystal is positioned with its *XZ* plane on the bottom (scheme (1) in Figure 1b), the observation direction of microscopy is along the crystal *Y* coordinate. The primary data provided by interference microscopy are therefore, except for a constant, unknown factor of proportionality, the changes in the concentration integral:

(1) $$C_{Y}\left(X,Z\right)=\int_{Y=0}^{Y=L_{Y}}c\left(X,Y,Z\right)dY$$

or, for constant crystal thickness completely equivalently, the mean concentration <*c*(*X*, *Z*)>*_Y_* along this direction. We have used the notations *c*(*X*, *Y*, *Z*) for the local concentrations at positions *X*, *Y*, *Z* of a given crystal and *L_Y_* for the crystal thickness in *Y* direction. In the plane perpendicular to the observation direction, the concentration integral or the mean concentration (in the case referred to as *C_Y_*(*X*,*Z*) or <*c*(*X*, *Z*)>*_Y_*, respectively) may be determined with a spatial resolution (∆*X*, ∆Z) of about 0.5 μm.

**Figure 2:**
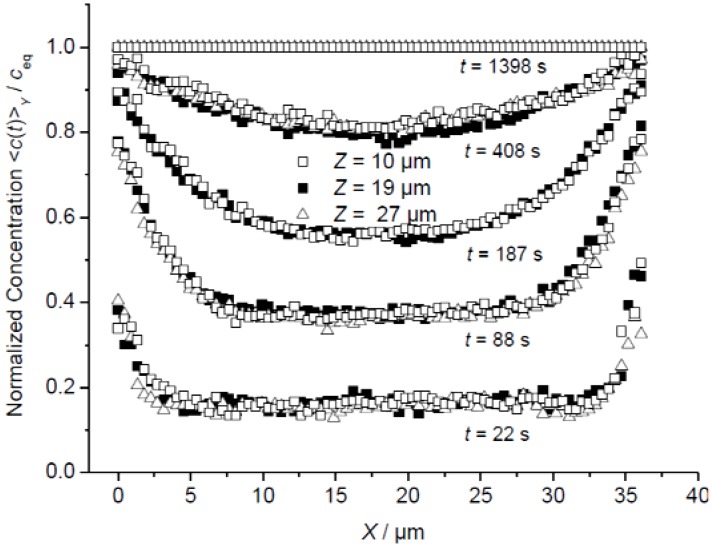
Comparison of the evolution of the profiles of mean intracrystalline concentration perpendicular to the *XZ* plane (mean concentrations in *Y* direction), in *X* direction, at different locations *Z* (□, ■, Δ, see Figure 1b, scheme 1) during molecular uptake of methyl-butane in silicalite.

**Figure 3:**
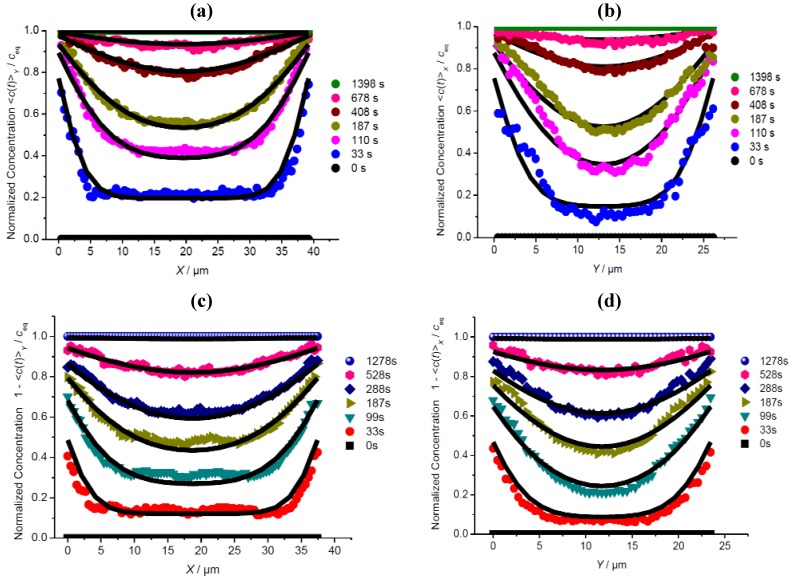
Profiles of mean concentrations during uptake (**a**,**b**) and release (**c**,**d**) of methyl-butane observed perpendicular to the *XZ* plane along *X* (**a**,**c**, Figure 1b, Scheme 1, central line) and perpendicular to the *YZ* plane along *Y* (**b**,**d**, Figure 1b, Scheme 2) and comparison with the solution of Fick’s 2nd law with the diffusivities and surface permeabilities given in Table 1 (solid lines).

**Figure 4:**
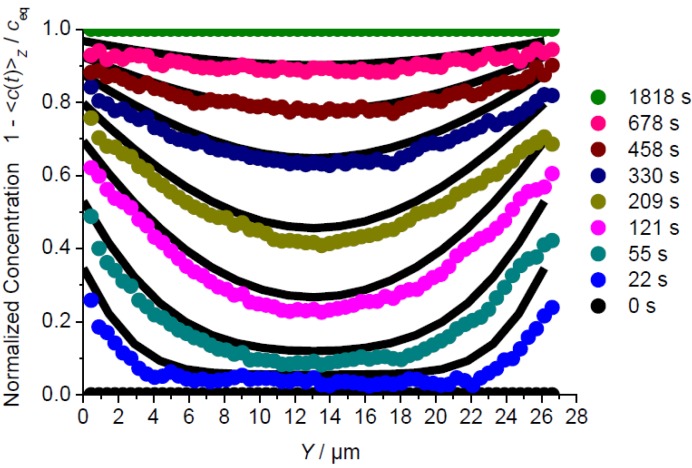
Evolution of the profiles of mean concentration <*c*(*t*)>*_z_* determined by observation perpendicular to the *XY* plane along the *Y* direction (Figure 1b, Scheme 3) during release of methyl-butane by a pressure step from 1 mbar to 0. The experimental values (circles in color) are complemented by the data determined from the solution of Fick’s 2nd law with the diffusivities and permeabilities given in Table 1 (black lines).

**Figure 5:**
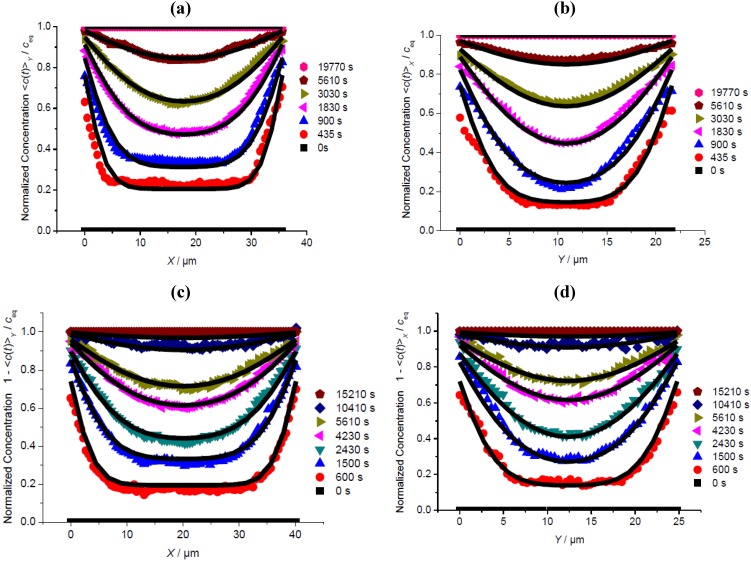
Profiles of mean concentrations during uptake (**a**,**b**) and release (**c**,**d**) of benzene observed perpendicular to the *XZ* plane along *X* (**a**,**c**, Figure 1b, Scheme 1, central line) and perpendicular to the *YZ* plane along *Y* (**b**,**d**, Figure 1b, Scheme 2) and comparison with the solution of Fick’s 2nd law with the diffusivities and surface permeabilities given in Table 1 (solid lines).

**Figure 6:**
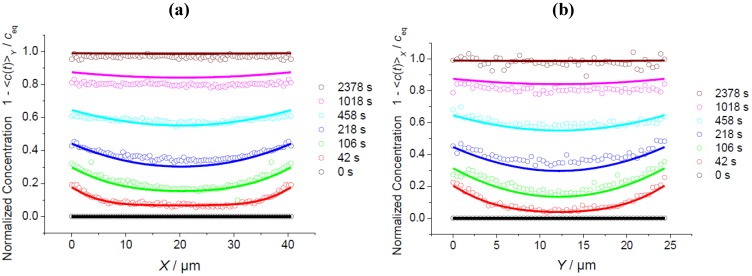
Profiles of mean concentrations during release of 4-methyl-2-pentyne observed perpendicular to the *XZ* plane along *X* (**a**, see Figure 1b, Scheme 1, central line) and perpendicular to the *YZ* plane along *Y* (**b**, Figure 1b, Scheme 2) and comparison with the solution of Fick’s 2nd law with the diffusivities and surface permeabilities given in Table 1 (solid lines).

Instead of representing the evolution of the measured concentration integral over the whole plane of observation (*i.e.*, in the selected case, of *C_Y_*(*X*,*Z*,*t*) or <*c*(*X*, *Z*)>*_Y_* over the complete *XZ* plane), for the sake of clarity we confine ourselves to cross-sections through the “landscape” of concentration integrals, *i.e.*, to plotting the profiles along only certain lines. The straight lines shown in Figure 1b indicate the lines along which the concentration integrals shown in Figure 2, Figure 3, Figure 4, Figure 5 and Figure 6 have been determined.

To obtain the diffusivities and permeabilities in different directions *X*, *Y*, *Z* (see Figure 1b), the crystal under study must be flipped at least once, with exactly the same adsorption and desorption cycle being applied in each orientation. Due to the curved shape of the corresponding crystal face, (Figure 1b) experiments recording the concentration profiles in the *XY*-plane are particularly demanding. In addition to the instability of this crystal position, the analysis of the concentration profiles is also complicated by the varying crystal thickness *L_Z_* and the occurrence of light diffraction and scattering. Therefore, except for Figure 4 which demonstrates the feasibility of investigating the evolution of concentration profiles in this *XY*-plane for methyl-butane, we confined ourselves to the measurement of transient profiles in the *XZ*- and *YZ*-planes.

To correlate the measured concentration integrals with the transport parameters giving rise to the observed behavior, the general solution of the appropriate form of the diffusion equation (Fick’s 2nd law) for a model crystal is used, including surface resistances and the corresponding boundary conditions. In crystal *X* direction, e.g., the corresponding relations are:

(2) $$\dot{c}\left(X,Y,Z,t\right)=\frac{\partial }{\partial X}\left(D_{X}\frac{\partial }{\partial X}c\left(X,Y,Z,t\right)\right)$$

and

(3) $$D_{X}\frac{\partial }{\partial X}c{\left(X,Y,Z,t\right)}_{X=0(L_{X})}=\;\alpha_{X}\left[c\left(X=0(L_{X}),Y,Z,t\right)-c\left(\infty \right)\right]$$

where we have assumed that the particle under study may be considered to behave as a single crystal. The diffusivity (*i.e.*, the principal tensor element) in crystal *X* direction is *D_X_* and *α_X_* denotes the permeability through the two crystal faces perpendicular to the *X* direction (which are assumed to coincide). Further on, notably for large pressure steps covering a large range of intracrystalline concentration during uptake (release), both the diffusivities and the surface permeabilities may need to be considered as concentration dependent. For this purpose, the two-parameter equations

*D*(*c*) = *D_c_*_=0_ (1+ *c^a^*), *α*(*c*) = *α_c=0_* (1+ *c^b^*) (4)

are found to provide a useful approximation.

The relevant parameter set is determined from the best fit of the model calculations to the measured concentration integrals, in particular to their time dependence as observed over different pressure steps, including uptake and release. Clearly, only a small fraction of the parameters can be determined with sufficient accuracy by such a procedure but the parameters that can be found are exactly those that control the mass transfer rates in different directions.

### 2.3. The Guest Molecules under Study

The measurements have been performed with benzene, methyl-butane and 4-methyl-2-pentyne as guest molecules. Benzene is one of the “guinea pigs” used in numerous diffusion studies with MFI-type zeolites [41,68,69,70,71,72,73,74,75,76]. In contrast to n-alkanes where the diffusivities obtained by different research groups reveal order-of-magnitude differences (see reference [8], Section 18.2.1), the benzene diffusivities obtained in these studies are essentially similar, yielding, at room temperature, corrected diffusivities of ≈ 1 … 6 × 10^−14^ m^2^ s^−1^ (see reference [8], Section 18.3.1).

In previous IFM (interference microscopy) diffusion studies [58] methyl-butane was found to serve as an excellent probe molecule for the recording of transient concentration profiles. For crystal sizes typically about 10 μm, the measured intracrystalline diffusivity of 1–3 × 10^−13^ m^2^ s^−1^, *i.e.*, about one order of magnitude greater than the benzene diffusivities, turned out to allow the recording of several subsequent, still well-distinguished concentration profiles during molecular uptake and release, with minimal expenditure of time. As a consequence of the limited time resolution (20 seconds per profile under the given conditions), with any further increase of the diffusivity (e.g., with methyl-propane [58]) the number of profiles which may be recorded during uptake or release is reduced.

While both benzene and methyl-butane are bulky molecules which are expected to propagate by a jump-like movement between the channel intersections, the third probe molecule, 4-methyl-2-pentyne, is of rod-like structure. Following the investigations by Rees *et al.* [68,69], the diffusion of rod-like molecules in the straight channels of MFI-type zeolites is expected to be notably faster than in the sinusoidal channels. It is for this reason that 4-methyl-2-pentyne was included in our studies.

## 3. Results and Discussion

The measurement results are presented as selected (1D) concentration profiles through the three possible different planes of observation, taken along the lines shown in Figure 1b. The data points are compared with the corresponding solutions of Fick’s 2nd law, Equation 2, with the boundary condition, Equation 3. The full lines represent the solutions yielding the best fit to the experimental data points, determined under the simplifying condition of constancy of the transport parameters *D* and *α*. Table 1 provides a summary of these values.

**Table 1:** Diffusion coefficients *D* and surface permeabilities *α* at room temperature (≈ 295 K) for methyl-butane, benzene and 4-methyl-2-pentyne as guest molecules in the rounded-boat-shaped crystals of silicalite considered in this study. The given numbers are obtained from best fits of the solution of Fick’s 2nd law to the experimental data (full lines in Figure 2, Figure 3, Figure 4, Figure 5 and Figure 6), determined separately for adsorption and desorption under the assumption of a negligibly small concentration dependence of *D* and *α*. Except for 4-methyl-2-pentyne, the diffusivities and surface permeabilities in *X* and *Y* direction are assumed to coincide.

	Ads/Des, pressure (mbar)	Direction	*D* (m^2^ s^−1^)	*α* (m s^−1^)
**Methyl-butane**	Ads, 0–1	*X*,*Y*	2.4 × 10^−13^	1.5 × 10^−7^
	Des, 1–0	*X*,*Y*	2.6 × 10^−13^	5.0 × 10^−8^
**Benzene**	Ads, 0–0.5	*X*,*Y*	1.5 × 10^−14^	9.5 × 10^−9^
	Des, 1–0	*X*,*Y*	1.4 × 10^−14^	7.0 × 10^−9^
**4-Methyl-2-pentyne**	Des, 1–0	*X*	4.4 × 10^−13^	1.9 × 10^−8^
	Des, 1–0	*Y*	5.2 × 10^−13^	1.3 × 10^−8^

### 3.1. Mass Transfer in Z Direction

Figure 2 shows the evolution of integral guest profiles during the uptake of methyl-butane during a pressure step from 0 to 1 mbar determined by observation perpendicular to the *XZ* plane in *X* direction, for three different values of *Z.* The profiles are found to coincide for different values of *Z*. Mass transfer in *Z* direction may therefore be excluded. Otherwise, the profiles at *Z* = 10 and 27 μm (*i.e.*, close to the crystal faces at *Z* = 0 and *L_Z_*) should attain equilibrium notably faster than the central profile (at *Z* = 19 μm).

On the basis of these measurements it cannot be determined whether the absence of any significant mass transfer in the *Z* direction is caused by a large surface resistance on the relevant crystal faces (*Z* = 0 and *L_Z_*) or by a dramatically reduced diffusivity in this direction. The latter explanation would be in agreement with the supposition that the straight and sinusoidal channels extend preferentially in the *XY* plane. In this case, with the correlation rule of diffusion anisotropy in MFI-type zeolites [26,27], the diffusivities in *Z* direction are indeed found to be much smaller than in *X* and *Y* direction.

### 3.2. A Check of Diffusion Anisotropy in the XY Plane

Figure 3 compares the profiles of transient integral concentrations during molecular uptake and release, recorded along the central line in the *X* direction in the *XZ* plane (Figure 1b, Scheme 1) and in the *Y* direction in the *YZ* plane (Figure 1b, Scheme 2). To facilitate the direct comparison of the transient concentration profiles during uptake and release, the (normalized) concentrations <*c*(*t*)>/*c*_eq_ during release are plotted as 1–<*c*(*t*)>/*c*_eq_.

Since, with the measurements described in Section 3.1, any significant mass transfer in the *Z* direction may be excluded, profile evolution in these representations is easily seen to be exclusively due to mass transfer in the *X* and *Y* directions, with the observation direction either perpendicular to *X* (a,c) or *Y* (b,d). Differences in the diffusivities in the *X* and *Y* directions should therefore be revealed by this type of comparison particularly clearly.

On comparing the experimental data shown (Figure 3(a–d)) with the analytical expressions from the solution of Fick’s 2nd law, it turned out, however, that the assumption of coinciding diffusivities in the *X* and *Y* directions (see Table 1) leads to excellent fits which cannot be further improved by assuming diffusion anisotropy with respect to the *X* and *Y* directions. On comparing the transient profiles in the *X* (left column) and *Y* (right column) directions one should not be disturbed by the different shapes: this arises simply as a consequence of the different crystal dimensions in these directions.

The slight differences in the fitting parameters *D* and *α* for the adsorption and desorption runs may be easily attributed to the simplifying assumption of their concentration independence. In fact, the transient concentration profiles during both adsorption and desorption are found to be completely satisfactorily approximated with a constant value of 2 × 10^−13^ m^2^ s^−1^ for the diffusivity and a permeability *α* (*c*) = 0.8 (1+ *c*^2^) × 10^−7^ m s^−1^, following the concentration pattern of Equation 3, as a first-order approach. Over the (normalized) concentration, the surface permeability is thus found to vary from 0.8 to 1.6 × 10^−7^ m s^−1^. This factor of 2 between the largest and smallest diffusivity in the concentration range may, quite generally, be assumed as a measure of the accuracy of the determined diffusivities and surface permeabilities. The fact that the transport parameters *D* and *α* do not depend significantly, if at all, on concentration is revealed already by the similar shapes of the intracrystalline concentration profiles during uptake (top of Figure 3) and release (bottom of Figure 3).

As an example of transient sorption experiments recorded by observation along the third direction, Figure 4 shows the profiles during methyl-butane release under the conditions considered in Figure 3b,d. The curvature of the crystal surface perpendicular to observation direction gave rise to a much lower accuracy of the profiles. However, also in this case, the values determined from the analytical solution of Fick’s 2nd law with the data given in Table 1 (black full lines) are found to satisfactorily approximate to the measured values.

### 3.3. Comparison of Different Guest Molecules

In addition to the transient concentration profiles during uptake and release using methyl-butane as a guest molecule (Figure 3), Figure 5 and Figure 6 provide corresponding plots for benzene and 4-methyl-2-pentyne. The diffusivities and surface permeabilities used to obtain the best fits between the calculations (full lines) and the experimental data points are listed in Table 1. The message from the benzene data (Figure 5) is identical to the information provided already by using methyl-butane as a probe molecule: There is no perceivable anisotropy with respect to the *X* and *Y* directions in either the diffusivities or the surface permeabilities. It is interesting to note that both the diffusivities and surface permeabilities for benzene are about one order of magnitude smaller than for methyl-butane. This is nicely reflected by the similarity of the concentration profiles, with a shift in the time scales by about this order of magnitude for benzene in comparison with methyl-butane.

Owing to its rod-like structure, 4-methyl-2-pentyne is assumed to trace differences in the diffusivities along the straight and sinusoidal channels with a much higher sensitivity than the more bulky molecules methyl-butane and benzene [68,69]. Hence, with this molecule, the best fit resulting from the solution of Fick’s 2nd law to the experimental data was determined by varying the diffusivities and surface permeabilities in *X* and *Y* independently from each other. The resulting data for the *X* and *Y* directions given in Tab. 1 are seen to coincide for both the diffusivities and surface permeabilities within the limit of accuracy. It thus appears that none of the considered molecules provide any evidence of significant diffusion anisotropy in the rounded-boat-shaped MFI-type crystals used in this study. This result that is consistent with the conclusions from structural analysis of a series of MFI-type crystals [59,60,77] and implies that the structures of both the coffin-shaped and boat-shaped MFI crystals [61] are generally not crystallographically coherent.

### 3.4. Impact of Surface Resistances

Comparison of the diffusivity and permeability data for the three guest molecules under study in Table 1 reveals a remarkable peculiarity of 4-methyl-2-pentyne: While its surface permeability is similar to that of benzene, the intracrystalline diffusivities exceed those of benzene by an order of magnitude. Hence, while for methyl-butane and benzene the relative importance of intracrystalline diffusion and surface permeation were found to be comparable (leading to essentially coinciding transient profiles as discussed above), molecular uptake and release of 4-methyl-2-pentyne is affected much more significantly by the mass transfer resistance at the crystal surface. This difference appears immediately in the different shapes of the respective concentration profiles shown in Figure 5 and Figure 6.

**Figure 7:**
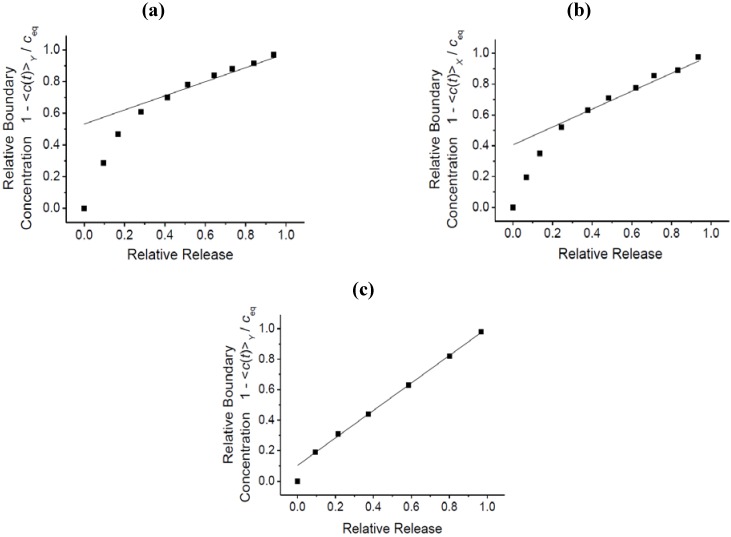
Relative boundary concentration as a function of relative release for benzene, calculated from the transient concentration profiles (**a**) in the *X* direction (shown in Figure 5c, area under the respective profiles; and (**b**) in the *Y* direction (shown in Figure 5d), and (**c**) for 4-methyl-2-pentyne (shown in Figure 6a).

Taking advantage of the novel options of micro-imaging of transient concentration profiles, Heinke and Kärger [65,78,79,80] suggested a special type of plot to show the relevance of surface resistances on the overall rate of molecular uptake and release and to provide immediate quantification. In these plots, by considering the relative boundary concentration as a function of relative uptake (or release), any time dependence is eliminated from the presentation. Examples of such curves are shown in Figure 7. They were determined from the transient concentration profiles shown in Figure 5 and Figure 6.

The ordinate intercept *w* can be shown to provide an estimate of the ratio *τ*_diff_/*τ*_diff+surf-barr_ between the mean time of molecular uptake through the given crystal face (face perpendicular to observation direction) if there were no surface resistance at all (*τ*_diff_ = *L*^2^/12*D*) and the time constant under the combined influence of surface permeation and intracrystalline diffusion (*τ*_diff+surf-barr_ = *τ*_diff_ + *τ*_surf-barr_ = *L*^2^/12*D* + *L*/2*α*) [8,80].

From the ordinate intercept in Figure 7c (*w* ≈ 0.1), for 4-methyl-2-pentyne the transport resistance by the surface barrier is seen to exceed the diffusion resistance by one order of magnitude while, from the intercepts in Figure 7a (*w* ≈ 0.5) and 7b (*w* ≈ 0.4), for benzene these two resistances are found to be similar. In fact, the slightly smaller value of *w* ≈ 0.4 observed with respect to the shorter crystal extension (Figure 7b), corresponding to a slightly enhanced influence of the surface resistance, is to be expected, since the diffusional resistance increases with the square of the extension *L* of the crystal.

Following the reasoning of refs. [48,50], the similarity of the diffusivity/permeability ratios for methyl-butane and benzene may be taken as an indication that the surface resistance is caused mainly by a total blockage of most of the entrance pores, with a few holes allowing essentially unrestricted passage. For 4-methyl-2-pentyne data, however, the impediment of mass transfer through the crystal surface is found to be much more pronounced. The presently available data do not allow any specification of the possible reasons leading to such behavior.

## 4. Conclusions

Interference microscopy has been shown to yield new insights into mass transfer behavior of guest molecules in nanoporous crystals. The quantity directly accessible (except for an unknown factor of proportionality) is the concentration integral (or, completely equivalently, the concentration average) in observation direction. By recording the evolution of these profiles one can determine the intracrystalline diffusivities and the surface permeabilities for the directions and crystal faces considered.

Using a certain type of crystals of zeolite silicalite (so-called rounded-boat-shaped crystals), in the present studies, for the first time, transient concentration profiles in MFI-type zeolites could be recorded by observation in all three directions (*i.e.*, with the crystals positioned on each of the three different crystal faces). The experimental results obtained with the probe molecules under study (methyl-butane, benzene and 4-methyl-2-pentyne) include the observation of dramatically decreased mass transport in the crystal *Z* direction and the absence of any perceptible difference in the diffusivities along the crystal *X* and *Y* directions. At least the rod-like 4-methyl-2-pentyne molecules would be expected to show notable differences in the diffusivities along the straight and sinusoidal channels of MFI structure. The absence of any diffusion anisotropy in the crystal *X* and *Y* directions must therefore be taken as an indication that the rounded-boat-shaped MFI-type crystals under study are intergrowths rather than single crystals as has been found generally for other forms of MFI.

Comparison of the intracrystalline diffusivities and surface permeabilities determined in these studies shows a remarkable peculiarity. While for methyl-butane and benzene the transport resistance on the crystal surfaces (*XZ* and *YZ* planes) may be expected to be caused by essentially total blockage of the vast majority of the pore entrances on the crystal surface, with only a few pores being permeable, surface permeation of 4-methyl-2-pentyne appears to follow a completely different mechanism, leading to an additional reduction in permeability. The exploration of the origin of these differences is among the challenging new questions emerging from the application of interference microscopy to the study of mass transfer phenomena in nanoporous materials.
